# Veiled Dangers in an Idyllic Setting

**DOI:** 10.3201/eid2602.AC2602

**Published:** 2020-02

**Authors:** Byron Breedlove

**Affiliations:** Centers for Disease Control and Prevention, Atlanta, Georgia, USA

**Keywords:** art science connection, emerging infectious diseases, art and medicine, about the cover, veiled dangers in an idyllic setting, A Rajput Warrior with Camel, Possibly Maru Ragini from a Ragamala, public health, camel, ragmala, viruses, coronaviruses, Middle East respiratory syndrome, MERS, Middle East respiratory syndrome coronavirus, MERS‐CoV, brucellosis, Echinococcus granulosus, Rift Valley fever, severe acute respiratory syndrome, (SARS), zoonoses

**Figure:**
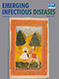
**Artist Unknown. A Rajput Warrior with Camel, Possibly Maru Ragini from a Ragamala, 1650–80. Opaque watercolor, ink, and gold on paper.** Ten 1/4 in x 6 15/16 in/26 cm x 17.6 cm. Purchase and partial gift from the Catherine and Ralph Benkaim Collection; Severance and Greta Millikin Purchase Fund. Public domain digital image courtesy of The Cleveland Museum of Modern Art, Cleveland, Ohio.

The modern word camel is derived from the Latin word *camelus*, the Greek word *kamēlos*, and the Hebrew word *gāmāl*, which means “going without,” in reference to the camel’s ability to survive and function without food or water for days. Camels can “go without” thanks in large measure to their humps, which contain up 80 pounds of fat that can be broken down into water and energy. This onboard reservoir enables them to travel up to 100 miles through the desert without water. Several other adaptations help camels thrive in dry, desert environments, where temperatures can soar to more than 100°F in summer and plummet to –20°F or colder in winter.

For thousands of years, the people of North Africa and Asia have used the one-humped Arabian camels of the species *Camelus dromedarius* (commonly called dromedaries) for transporting people and conveying goods essential for trade and sustenance. Dromedaries, one of the largest species of domesticated livestock, may reach a shoulder height of 7 feet, and the males typically weigh between 900 and 1,300 pounds. More than serving as beasts of burden, they have also sustained human societies living in inhospitable environments as a source of meat, milk, and leather.

This month’s cover art, *A Rajput Warrior with Camel, Possibly Maru Ragini from a Ragamala*, celebrates the longstanding relationship between humans and camels. The painting comes from India, the country at the eastern end of the range for this type of camel. Within India, these camels are concentrated in the northwestern state of Rajasthan, and smaller populations are found in the neighboring states of Gujarat and Haryana

*A Rajput Warrior with Camel is* an example of ragamala painting, a genre that emerged in medieval India. According to the Metropolitan Museum of Modern Art, “A ragamala, translated from Sanskrit as ‘garland of ragas,’ is a series of paintings depicting a range of musical melodies known as ragas. Its root word, raga, means color, mood, and delight, and the depiction of these moods was a favored subject in later Indian court paintings.” In many cases the mood, or raga, is written as poetry on the margins of the painting, and such works “express the intersections of painting, poetry, and music in Indian court art.” A ragamala typically included 36 or 42 loose-leaf paintings collected in a portfolio by members of various court circles who commissioned the work.

The young warrior portrayed on the painting balances on a fallen tree trunk; his domesticated dromedary compliantly kneels before him. He holds a thin spear in his right hand and grasps a halter in his left. In addition to his spear, he has a dagger inserted into the sash around his waist and a gold-handled scimitar in a gold-tipped scabbard. Despite this trio of weapons, or perhaps because of them, the young man appears confident and relaxed. He is resplendently garbed in finery from head to toe. The accoutrements and attention to details also accorded to his camel―a decorative bridle, ornate fabric covering its hump, a plumed headdress, and brocade cords―signal privilege and refinement.

The prevailing mood of this scene is benevolence and peace between human and animal. The lush setting adds to the sense of calmness. Pairs of whorled evergreens and broad-leafed tropical trees fill the upper half of the image. Billowing clouds hover over the gentle slope of a hill, and several flowering plants appear throughout. Adding to the restive atmosphere is the bright orange border festooned with an ornate flower motif. If any danger is present, certainly it is far away and not of immediate concern to the warrior or camel.

Perhaps only an epidemiologist or virologist might spot veiled dangers in such an idyllic setting—such as the zoonotic diseases brucellosis and Rift Valley fever―that dromedaries may spread to humans. Some might think of other novel camel-borne diseases, including a prion disease recently found in camels at a slaughterhouse in Algeria. 

But the camel-borne zoonotic disease of most concern is a disease that emerged only in the past decade, Middle East respiratory syndrome (MERS ). This viral respiratory illness is caused by Middle East respiratory syndrome coronavirus, or MERS‐CoV, initially identified in Saudi Arabia in 2012. Dromedary camels are a reservoir host for this zoonotic virus, and researchers believe that the original reservoir hosts may have been bats because similar coronaviruses have been found in bats. More than a third of reported cases have resulted in death. Coronaviruses are a large family of viruses, many of which can cause diseases in humans, ranging from the common cold to severe acute respiratory syndrome (SARS). According to an article published in 2018, researchers also identified dromedary viruses related to human coronavirus (HCoV) 229E, a primarily nonlethal coronavirus that causes upper and lower respiratory tract infections in humans. These dromedary viruses could replicate in human cells, thus suggesting that HCoV-229E may have descended from camelid-associated viruses.

Almost all modern camels are domesticated, and their population is growing at a faster rate than that of other domestic livestock such as cattle, horses, sheep, and llamas (though at a lower rate than that for goats). Zoonotic infections such as MERS remind us that for all the shared benefits of long-term human and animal relationships, these close interactions between animals of different species can also yield unwanted consequences and require new protocols and approaches to mitigate disease transmission.
